# Genetic Panel Test of Double Cancer of Signet-Ring Cell/Histiocytoid Carcinoma of the Eyelid and Papillary Thyroid Carcinoma: Case Report and Literature Review

**DOI:** 10.7759/cureus.25192

**Published:** 2022-05-21

**Authors:** Masashi Kuroki, Hirofumi Shibata, Bunya Kuze, Toshimitsu Ohashi, Keishi Kohyama, Hisakazu Kato, Hiroki Kato, Tatsuhiko Miyazaki, Hiroyuki Tomita, Takenori Ogawa

**Affiliations:** 1 Otolaryngology, Gifu University Graduate School of Medicine, Gifu, JPN; 2 Otolaryngology, Kizawa Memorial Hospital, Gifu, JPN; 3 Plastic and Reconstructive Surgery, Gifu University Graduate School of Medicine, Gifu, JPN; 4 Plastic and Reconstructive Surgery, Gifu University Hospital, Gifu, JPN; 5 Radiology, Gifu University Graduate School of Medicine, Gifu, JPN; 6 Department of Diagnostic Pathology, Gifu University Hospital, Gifu, JPN; 7 Tumor Pathology, Gifu University Graduate School of Medicine, Gifu, JPN

**Keywords:** cadherin 1, genetic panel test, double cancer, papillary thyroid carcinoma, eyelid, signet-ring cell/histiocytoid carcinoma

## Abstract

Signet-ring cell/histiocytoid carcinoma (SRCHC) is a rare, aggressive neoplasm that often originates in the eyelid. We present a rare case of a 64-year-old male with SRCHC and papillary thyroid carcinoma (PTC) that underwent exome panel sequencing with next-generation sequencing (NGS). In addition, we reviewed reports of genetic mutations in SRCHC and compared them with our results. The imaging findings allowed us to recognize the differences in pathology between the left and right cervical nodes. For first-line treatment, an extended total maxillectomy with orbital exenteration and dissection of the left neck was performed. Two months later, total thyroidectomy and right neck dissection were performed. Two years after surgery, multiple bone metastases occurred. An exome panel sequence with NGS was used to determine the chemotherapy regimen. Notably, somatic mutations in cadherin 1 (CDH1), human epidermal growth factor receptor 2 (ERBB2), neurofibromin 1 (NF1), and tumor protein p53 (TP53) were detected. These mutations are rarely detected in PTC; therefore, cervical metastases are assumed to originate from SRCHC. To our knowledge, there have been no reports of simultaneous cancer of SRCHC and PTC. Somatic mutations in CDH1, ERBB2, NF1, and TP53 were detected in the exome panel sequence of the metastatic lymph nodes of SRCHC and correlated with previous reports of SRCHC.

## Introduction

Signet-ring cell/histiocytoid carcinoma (SRCHC) is a rare, aggressive adnexal neoplasm that preferentially affects the eyelid and histopathologically resembles a metastatic lobular carcinoma of the breast and/or some adenocarcinomas that arise in the gastrointestinal tract [1]. According to the World Health Organization (WHO) classification of skin tumors, appendageal tumors are classified as malignant tumors with apocrine and eccrine differentiation [2]. The SRCHC is mainly observed in the eyelids as the primary region, followed by the axilla [3]. Most of the reported patients presented with swelling of the eyelids as an initial symptom, and the eyelids gradually and diffusely thickened. Because it is quite characteristic of the tumor, it is called a “monocle tumor” [4]. In a case with bilateral eyelid swelling, it is also referred to as a “binocle tumor” [5]. The diagnosis of SRCHC requires the exclusion of metastases from other sites, especially the breast, stomach, and urinary tract. Histopathological and immunohistological examinations are essential for a definitive diagnosis. Tumor cells have vacuolated cytoplasm in the signet-ring cell variant or exhibit eosinophilic granular cytoplasm in the histiocytoid variant. Both types of cells can be found in a single lesion, but the predominant cell type exists in most cases [2]. Immunohistochemically, neoplastic cells express pan-cytokeratins such as AE1/AE3, cytokeratin 7 (CK7), E-cadherin, carcinoembryonic antigen (CEA), epithelial membrane antigen (EMA), gross cystic disease fluid protein-15 (GCDFP15), and human milk fat globules [1]. Herein, we present a rare case of synchronous double cancer of the SRCHC of the eyelid and papillary thyroid carcinoma (PTC).

## Case presentation

**Figure 1:**
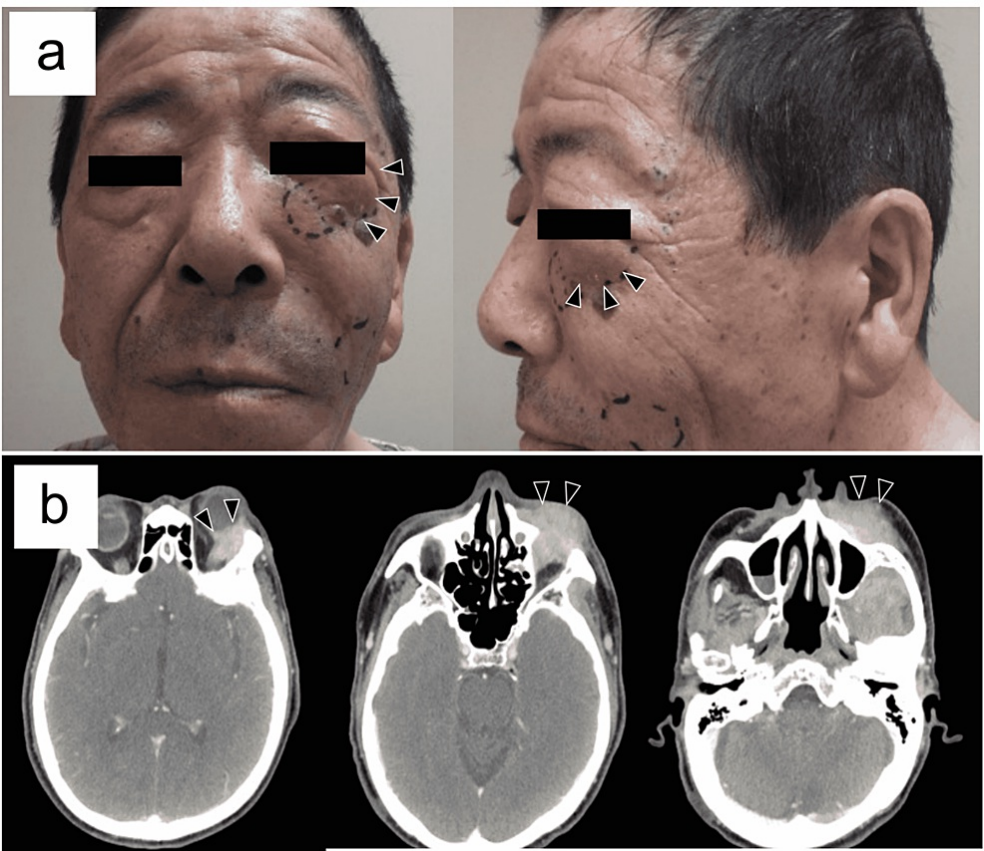
Preoperative and intraoperative findings a) Physical examination revealed swelling of the left internal canthus and cheek. Triangle lesions indicated signet-ring cell/histiocytoid carcinoma. b) Computed tomography showed an ill-defined mass in the left orbit, buccal skin, masticator space, and maxilla (arrowheads).

A 64-year-old male was referred to our hospital with a chief complaint of swelling of the left eyelid and cheek for a year. Physical examination revealed swelling of the left internal canthus and cheek, bilateral cervical lymphadenopathy, and a mass from the isthmus to the left lobe of the thyroid gland (Figure 1a).

**Figure 2:**
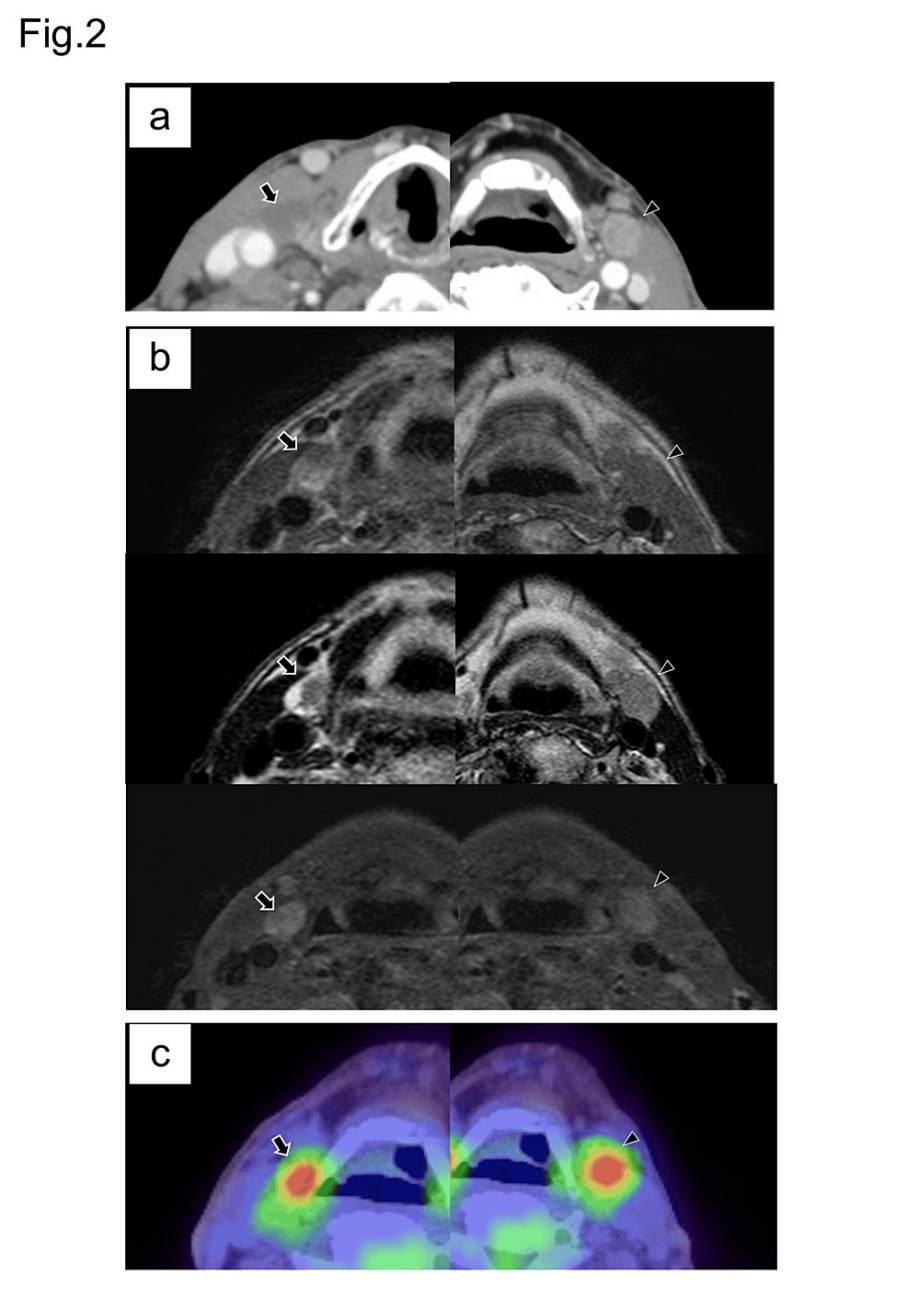
A comparison of imaging findings of the left and right lymph nodes a) Computed tomography showed a solid lymph node in the left neck (arrowhead) and cystic lymph nodes in the right neck (shown with arrows). b) Magnetic resonance imaging showed a focal hyperintense area in the T1-weighted images and a markedly hyperintense area in the T2-weighted images in the periphery of the right cervical node (shown with arrows). The left cervical nodes appeared as solid nodes with nonspecific signal intensity (indicated by arrowheads). The degree of contrast enhancement of the lymph nodes was greater in the right neck than in the left neck. c) Positron emission tomography/computed tomography showed no significant difference in fluorine-18 deoxyglucose accumulation between the right cervical nodes (shown with arrows) and the left cervical node (arrowhead).

Medical or family history was unremarkable. Histological examination of biopsy specimens obtained from a mass in the cheek indicated ductal carcinomas such as salivary duct carcinoma or signet-ring cell carcinoma. Computed tomography (CT) showed an ill-defined mass in the left orbit, buccal skin, masticator space, and maxilla (Figure 1b) with bilateral cervical lymphadenopathy. Multiple thyroid nodules were detected in both lobes. Irregular thyroid nodules with calcifications were observed in the isthmus. Magnetic resonance imaging (MRI) showed a left facial mass with hypointensity similar to skeletal muscle on T2-weighted images and mild hyperintensity on diffusion-weighted images. CT showed solid lymph nodes in the left neck and cystic lymph nodes in the right neck (Figure 2a).

MRI showed a focal hyperintense area on T1-weighted images and a markedly hyperintense area on T2-weighted images in the periphery of the right cervical node (Figure 2b). On contrast-enhanced MRI, the degree of contrast enhancement of the lymph nodes was greater in the right neck than in the left neck (Figure 2b). Positron emission tomography/computed tomography (PET-CT) did not show significant differences in fluorine-18 deoxyglucose accumulation between the right and left cervical nodes, and no distant metastases were observed (Figure 2c).

Based on the imaging findings, we decided that the primary lesions of the left and right cervical lymph nodes were different and diagnosed this case as a double cancer of SRCHC of the left eyelid (T4aN1M0, clinical stage IVa, according to the staging of carcinoma of the skin of the eyelid) and PTC (clinical T1bN1bM0, clinical stage II). We explained to the patient the differences in the primary lesions of the left and right cervical lymph nodes, surgery time, and the necessity of free flap reconstruction, and we performed two-stage surgery at the patient’s request. Because SRCHC was considered to have a higher malignancy than PTC, we first performed a left extended total maxillectomy with orbital exenteration, left neck dissection (levels I to IV), and reconstruction with a rectus abdominis musculocutaneous flap. The surgery was performed with an incision similar to the Weber-Ferguson incision and a left cervical arc incision. Since skin infiltration was observed in a wider area than expected before the operation, the skin was resected together (Figure 1d). After two months of follow-up, we performed a total thyroidectomy and right neck dissection (levels II to V).

**Figure 3:**
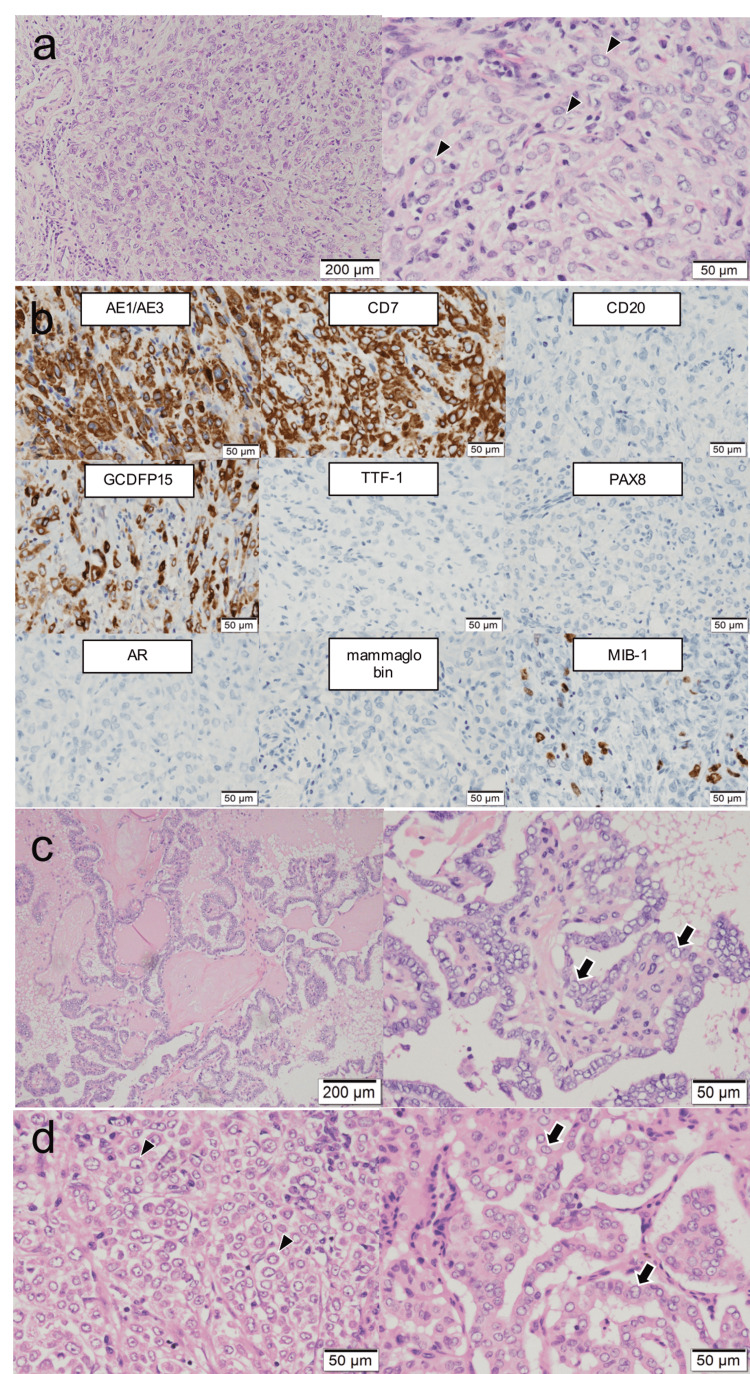
Postoperative pathological findings a) Postoperative histopathological findings showed a glandular malignant tumor with strong mucus formation and a signet-ring cell-like tumor with an uneven distribution of nuclei (indicated by arrowheads). b) Immunohistostaining findings showed cytokeratin (CK) AE1/AE3 (+), CK7 (+), CK20 (-), gross cystic disease fluid protein (GCDFP)15 (+), thyroid transcription factor (TTF)1 (-), paired-box-containing (PAX) 8 (-), androgen receptor (AR) (-), mammaglobin (-), and MIB-1 (10-20%). c) In both lobes of the thyroid gland, tumor follicles with ground glass nuclei and nuclear grooves infiltrate and proliferate in papillary, tubular, and follicular forms (shown with arrows). d) In the left cervical lymph node, cancer metastasis is found in 23 lymph nodes, all of which are signet-ring cell/histiocytoid carcinoma (arrowheads). In the right cervical lymph node, cancer metastasis is found in 26 lymph nodes, all of which are metastases of papillary thyroid carcinoma (shown with arrows).

Postoperative histopathological findings showed that the facial tumor was a malignant glandular tumor with significant mucus formation and a signet-ring cell-like tumor with an uneven distribution of nuclei (Figure 3a).

Immunohistostaining of the facial tumor showed cytokeratin (CK) AE1/AE3 (+), CK7 (+), CK20 (-), gross cystic disease fluid protein (GCDFP)15 (+), thyroid transcription factor (TTF)1 (-), paired-box-containing (PAX)8 (-), androgen receptor (AR) (-), mammaglobin (-), and MIB-1 labeling index was 10-20%. CK AE1/AE3 (+) suggests epithelial malignancy, and the combination of CK7 (+) and CK20 (-) suggests that the origin of the gastrointestinal tract was negative. Since TTF1 and PAX8 were negative, the tumor was believed to originate from other than the thyroid. AR and mammaglobin were negative, indicating that the tumor did not originate from the mammary gland. Based on these results, the pathologists diagnosed the tumor as SRCHC (Figure 3b). In both lobes of the thyroid gland, tumor cells with ground glass nuclei and nuclear grooves infiltrated and proliferated in papillary, tubular, and follicular forms (Figure 3c). Some vesicles are accompanied by sand grain formation, necrosis, and fibrous film formation, which are diagnosed as PTCs. In the left cervical lymph node, cancer metastasis was found in 23 lymph nodes, all of which were diagnosed as metastases of SRCHC (Figure 3d). In contrast, in the right cervical lymph node, metastasis was found in 26 lymph nodes, all of which were diagnosed as metastases of PTC (Figure 3d). These results showed that the postoperative staging of facial and thyroid tumors was diagnosed as pathological T4aN1 in SRCHC and pathological T4aN1b in PTC.

Two years after surgery, multiple bone metastases were found, and a radiologist performed a biopsy of the bone metastasis. Histopathological findings indicated metastasis from SRCHC. Since there is no standard chemotherapy regimen for metastatic SRCHC, next-generation sequencing (NGS) with FoundationOne®︎ CDx (Foundation Medicine, Cambridge, Massachusetts) was performed to determine the chemotherapy regimen. With the consent of the patient, NGS was performed on histological samples of metastatic lymph nodes of the SRCHC resected by primary surgery. Formalin-fixed paraffin-embedded tissue from metastatic lymph nodes was used for genomic testing of 324 cancer-related genes using FoundationOne CDx. The biomarker findings were microsatellite status-stable and six mutations/megabase for tumor mutational burden. Gene mutations were found in cadherin 1 (CDH1, E243K), human epidermal growth factor receptor 2 (ERBB2, D769Y), neurofibromin (NF1, K1171), and tumor protein p53 (TP53, H193L). However, no therapies with clinical benefits have been reported in this tumor type (Table 1). After all, we performed chemotherapy with a combination of pembrolizumab, cisplatin, and fluorouracil using receptor activator of nuclear factor kb ligand (RANKL) inhibitors. The therapeutic effect was evaluated after three courses of chemotherapy, but bone metastasis increased.

**Table 1:** Results of a next-generation sequencer (FoundationOne®︎ CDx) The biomarker findings were microsatellite status-stable and six mutations/megabase for tumor mutational burden. Gene mutations were found in cadherin 1 (CDH1, E243K), human epidermal growth factor receptor 2 (ERBB2, D769Y), heurofibromin (NF1, K1171), and tumor protein p53 (TP53, H193L).

Biomarker findings				
Microsateliites status			Microsatelites - stable	
Tumor mutation burden			6 mutations/megabase	
Genomic findings				
Gene	Alteration	Coding sequence effect	Variant allele frequency (%)	Therapies with clinical benefit
ERBB2	D769	2305G>T	0.92	none
NF1	K1171	3511A>T	10.7	none
CDH1	E243K	727G>A	16.4	none
TP53	H193L	578A>T	4.6	none

## Discussion

In this case, based on radiological and immunohistological examinations, the patient was diagnosed as having double cancer of SRCHC and PTC. As SRCHC is an extremely rare tumor, we searched PubMed for "signet-ring cell/histiocytoid carcinoma". We found only 13 articles, of which nine articles and 13 cases originated in the eyelid (Table 2) [1,5-12].

**Table 2:** Previous reports of signet-ring cell/histiocytoid carcinoma of the eyelid A total of 14 cases were presented, including the previously reported 13 cases and our case. It often occurs in elderly males and has a long disease duration of 6-24 months. Surgical treatment is performed in all cases described in detail, and recurrence is observed in six cases. M - male; F - female; ND - not described; OP - operation; PORT - postoperative radiation therapy; POCRT - postoperative chemoradiation therapy.

Author	Year	Age	Sex	Disease duration	Metastasis	Treatment	Recurrence	Genetic mutation
Requena L [1]	2011	65	M	24 months	No	OP+PORT	Yes	
		76	M	24 months	No	none	No	
		78	M	12 months	No	OP+PORT	No	
		59	F	12 months	No	OP+PORT	Yes	
		84	M	ND	No	OP	ND	
Iwaya M [9]	2012	72	M	6 months	No	OP+POCRT(S-1)	Yes	
Jantima T [12]	2013	74	M	ND	No	ND	No	
Bernárdez C [5]	2016	78	M	ND	No	ND	No	
Sakamoto K [6]	2017	74	M	ND	ND	OP	Yes	
Palakkamanil MM [7]	2020	73	M	24 months	No	OP+PORT	No	
Raghavan SS [8]	2020	85	M	18 months	Yes	OP+PORT+Tamoxifen	No	NTRK3,CDH1,CDKN1B,PIK3CA
Stewart S [10]	2020	84	M	24 months	ND	OP	Yes	
Jakobiec FA [11]	2020	88	M	6 months	No	OP+PORT	No	CDH1
Our case		64	M	12 months	Yes	OP	Yes	CDH1,ERBB2,NF1,TP53

Although SRCHC is clearly defined in the WHO classification, some similar cases have been reported, such as "signet-ring cell carcinoma of the eyelid", "histiocytoid carcinoma of the eyelid", and "apocrine carcinoma", suggesting that there are more cases of SRCHC diagnosed as different diseases names.

This tumor is most common in elderly males; in these 13 cases, the age of the patients ranged from 59 to 88 years, an average of 76 years, and 92% were male. The average time from the first symptom to the hospital visit was 18 months [1,5-12]. In this case, swelling of the left eyelid was the initial symptom, and the entire left face gradually swelled; he visited the hospital one year after the initial symptom.

Of the previously reported 13 cases (nine reports), three cases [6-8] were AR-positive, and two cases were estrogen receptor (ER) positive, which were treated with hormone therapy using bicalutamide and tamoxifen, respectively [8,9]. One case was positive for GATA3 [7], generally positive for breast cancer, bladder cancer, salivary gland cancer, etc. Extended resection and neck dissection are recommended for treatment. Except for a case that was not treated at the patient's request, all cases were surgically treated, 70% of patients underwent postoperative radiation therapy, and one case was combined with S-1. As mentioned above, there were some cases in which hormone therapy was performed postoperatively. In this case, extended resection and neck dissection were performed, and postoperative radiotherapy was not performed. The primary tumor site was controlled, although the patient was diagnosed with distant bone metastasis 18 months after the operation. Based on the fact that 43% of cases with postoperative radiation therapy had recurrent metastases (two local recurrences and one distant metastasis recurrence), complete resection is considered important for SRCHC due to the complex anatomical characteristics around the eyelid.

The SRCHC lesion did not show any characteristics of PTC, including ground glass nuclei, nuclear grooves, and TTF1/PAX8 expression, indicating that this case is a very rare double cancer. Focal hyperintense areas on T1-weighted images within the right cervical node allowed us to diagnose nodal metastases from PTC [13]. Alternatively, the degree of contrast enhancement was different between the left and right cervical nodes. These radiographical features contributed to recognizing the differences in pathology between the left and right cervical nodes.

Only one report analyzed SRCHC of the eyelid with next generation sequencing (NGS), in which neurotrophic receptor tyrosine kinase 3 (NTRK3), CDH1, cyclin dependent kinase inhibitor 1B (CDKN1B), and PIK3CA mutations were detected [8]. In this report, CDH1 (23.3%) had the highest variant allele frequency, consistent with our results (16.4%).

CDH1 (E243K) encodes the E-cadherin protein. The CDH1 (E243K) mutation generally occurs in other carcinomas with a signet-ring form, such as gastric cancer and breast cancer [14, 15]. CDH1 mutations promote the development of SRCHCs, including signet-ring cells.

TP53 is the gene most frequently detected in human cancer, and mutations have also been detected in 72% of head and neck squamous cell carcinomas [16]. In thyroid cancer, mutations in TP53 are detected in 30% of poorly differentiated cancers and 70% of undifferentiated cancers but are rare in papillary cancers [17].

The typical driver genes for PTC were not detected in this case. In other words, the developmental mechanisms of SRCHC and PTC generation were considered completely different in this case. Although SRCHC and PTC have relatively similar pathological findings, genetic testing can be used to distinguish between them. Based on our case and previous report, we summarized the differences between SHCRC and PTC in terms of driver gene mutations, histological characteristics, and radiological observations (Table 3).

**Table 3:** Difference between signet-ring cell/histiocytoid carcinoma (SRCHC) and papillary thyroid carcinoma (PTC) Summarized the differences between SHCRC and PTC in terms of driver gene mutations, histological characteristics, and radiological observations. SRCHC - signet-ring cell/histiocytoid carcinoma; PTC - papillary thyroid carcinoma; CDH1 - cadherin 1; CEA - carcinoembryonic antigen; EMA - epithelial membrane antigen; GCDFP15 - gross cystic disease fluid protein-15; AR - androgen receptor; ER - estrogen receptor; TTF1 - thyroid transcription factor 1; PAX8 - paired-box-containing 8

	SRCHC	PTC
Gene mutations	CDH1	BRAF, HRAS, KRAS, RET
Immunohistochemistry	E-cadherin, CEA, EMA, GCDFP15 (AR, ER, GATA3)	Thyroglobulin, TTF1, PAX8
Radiological observations of metastatic lymph nodes	solid lymph nodes T1: nonspecific T2: nonspecific contrast enhancement (+)	Cystic lymph nodes T1: focal hyperintense area T2: hyperintensity contrast enhancement (++)

Differentiated thyroid cancer has a good prognosis and progresses slowly, and is often observed in asymptomatic patients. According to a cohort study, 49% of thyroid cancers were accidentally found [18]. In addition, a meta-analysis reported that the prevalence of autopsy-detected thyroid cancers was >10% [19]. Therefore, in patients with thyroid cancer, it is likely to be a double cancer, but to our knowledge, there have been no reports of simultaneous SRCHC with PTC, and this case is very rare.

## Conclusions

We experienced a very rare case of double cancer of the SRCHC of the eyelid and PTC. Although both SRCHC and PTC are histologically similar cancers that both originate in glandular tissue, SRCHC showed histological characteristics not found in PTC, such as TTF1 and PAX8 negative. Based on pathological and imaging findings, the origins of the left and right lymph node metastases were considered different. Gene mutations in CDH1, ERBB2, NF1, and TP53 were found by NGS-based genetic panel tests and correlated with previous reports of SRCHC. There is no established treatment for SRCHC, and it is hoped that cases that include genetic findings will accumulate.
